# The Institutional Worker

**Published:** 1912-05-11

**Authors:** 


THE
### INSTITUTIONAL WORKER
**Being a Special Supplement to "The Hospital"**

### Editor's Notices.

**Contributions:**

Contributions should be written, or preferably typed, on one side of the paper only, and all articles sent in are accepted upon the distinct understanding that they are forwarded to The Hospital only.

The Editor cannot undertake to return MSS. not used, but when a stamped directed envelope is enclosed the MSS. may be returned if a special request is made.

Accepted articles and paragraphs of news will be paid for after publication at the scale rate.

**Address:**

To prevent delay all contributions and letters on editorial business must be addressed exclusively to the Editor, "The Hospital," 29 Southampton Street, Strand, London, W.C. It is important that this regulation shall be strictly observed.

**Correspondence:**

Correspondence on all subjects is invited. The name and address of correspondents must be given as a guarantee of good faith, but not necessarily for publication.

**Special Articles:**

Special articles are invited, and questions, inquiries, and paragraphs upon all matters relating to the work, administration, and management of general, special, mental, fever, cottage and convalescent hospitals, sanatoria, homes, institutions, societies and organisations for the treatment or care of the sick, injured and dependants of all classes. Every matter affecting the interests and welfare of the staff of all grades working in these institutions will receive special consideration.

**Administrative Medicine, Research and Items of News:**

Special terms will be paid for approved articles on subjects in this wide field by experts who have made some section of it their special study and interest. We wish to give prominence to every movement tending to advance accuracy of diagnosis, efficiency in treatment, and the development of modern methods to eradicate disease and promote the welfare of the suffering.

**A Bureau of Information:**

We invite inquiries and applications for information and help in providing for that numerous class of sufferers who require special care or house-room which they cannot obtain in their own homes or secure for themselves. **We cannot, however, prescribe, or recommend practitioners.**

**Books for Review:**

Publishers are particularly requested to send advance proofs of any new books of importance whenever possible, as the Editor has made arrangements to publish immediate reviews on a new plan.

**Photographs, Plans, Blocks, and Illustrations:**

It is requested that wherever possible MSS. may be accompanied by illustrations in any of the above forms. The name of the sender and of the article to which it belongs should be written on the back of each photograph, drawing, or block for purposes of identification.

**Local Papers:**

Newspapers containing reports or news paragraphs should be marked and addressed to the Sub-Editor.

**The Coupon System:**

A coupon will be found at the bottom of the third inside page of the cover each week. This coupon must be attached to every question or inquiry to which an answer is desired in The Hospital.

### Vacant Posts.

Every week a large and increasing number of important and lucrative appointments in all Departments of Institutional Life are announced in this section of The Hospital. As its name implies, this Supplement is devoted to the interests of the Institutional Worker, every effort being made to secure information for incorporation in this section of all Vacancies occurring in the Institutional, Poor-Law, and Municipal Services, and the especial attention of our readers is called to the undermentioned appointments, of which full particulars will be found in the following pages :—

| | Per Annum. |
|---|---|
| Cottage Hospital Matron | £50 |
| Theatre Sister | £35 |
| Charge Nurse | £34 |
| Children's Nurse | £30 |
| Cook-housekeeper | £40 |
| Two Certificated Nurses | £30 |
| Male Night Nurse | Salary 15s. per week. |

If you cannot find among the announcements a post suited to your experience and capabilities, there is the Appointments Wanted Column, which will enable you to bring your requirements directly to the notice of the Governing Bodies and Controlling Officials of Institutions of every description throughout the country for the small fee of 1s. for 21 words.

**Subscriptions,** which may commence at any time, are payable in advance. Cheques and Post-Office Orders should be crossed London County and Westminster Bank, Covent Garden Branch, and made payable to the Manager of The Hospital.

Rates of Subscription (payable in advance).

| UNITED KINGDOM. | | |
|---|---|---|
| Three Months (including Postage) | | 2s. 0d. |
| Six Months | do. | 4s. 0d. |
| Twelve Months | do. | 6s. 6d. |
| **FOREIGN AND COLONIAL.** | | |
| Three Months (including Postage) | | 2s. 6d. |
| Six Months | do. | 5s. 0d. |
| Twelve Months | do. | 8s. 8d. |

*SUBSCRIPTION FORM.*

*Please send me* The Hospital *for......................months, commencing with your issue of................................, for which please find enclosed............................*

*Name.................................................*

*Address ...................................................*

*...................................................*

*Date.....................................*

*To............................Newsagent.*

### Manager's Notices.

**All letters on business matters must be addressed to The Manager, "The Hospital," The Hospital Building, 28 and 29 Southampton Street, London, W.C.**

**Advertisements:**

To insure insertion, all advertisements for The Hospital must reach the Manager not later than Wednesday morning in each week. For scale of charges see pages vii and 2.

**Special Rates** will be quoted for those Institutions that agree to send all their vacancy, contract, and public notices to The Hospital each year, so as to make it a file of such announcements for ready reference.

**Remittances:**

**It is especially requested that remittances be made by cheque or Postal Order, and in halfpenny stamps only when the amount is under sixpence.**

### Appointments Vacant.

### TWO USEFUL HANDBOOKS FOR SECRETARIES.

HOSPITAL EXPENDITURE: THE COMMISSARIAT. For Hospital Matrons and Managers. 2/6, post free.

"This should prove a valuable handbook to those upon whom rests the responsibility of hospital finance, and should be of interest to the public as giving a fair idea of what this department should and does cost."—*Manchester Courier.*

A LEGAL HANDBOOK FOR THE USE OF HOSPITAL AUTHORITIES. By LEONARD SYER BRISTOWE, M.A. Oxon., Barrister-at-Law. 2/6, post free.

THE SCIENTIFIC PRESS, LTD.,
28 & 29 SOUTHAMPTON STREET, STRAND, LONDON, W.C.

### OFFICIAL APPOINTMENTS VACANT.

### Poor-Law Vacancies.

ASSISTANT LAUNDRESS (£25), Prestwich Union. Mr. E. W. Ogden, C. to G., Cheetham Hill Road, Manchester. May 13.

ASSISTANT LAUNDRESS (£20), Wirral Union. Mr. C. H. Spelman, Master of the Workhouse, Clatterbridge, near Birkenhead. May 15.

ASSISTANT MASTER (£35), Bristol Guardians. Mr. J. J. Simpson, C. to G., Bristol. May 13.

ASSISTANT TO MATRON (£22), Bridport Union. Mr. J. J. Roper, C. to G., Bridport. May 13.

ASSISTANT NURSE (£25), Solihull Union. F. L. Thompson, C. to G., Union Offices, Council House, Sparkhill, Birmingham. May 14.

ASSISTANT NURSE (£25), Macclesfield Union. Mr. F. May, C. to G., Macclesfield. May 13.

ASSISTANT NURSE (£22), Bridport Union. Mr. J. J. Roper, C. to G., Bridport. May 13.

ASSISTANT NURSE (£30), Easington Union. J. M. Longden, C. to G., Union Offices, Easington, Castle Eden. May 23.

ASSISTANT NURSE (£20), Kingsclere Union. Mr. J. Barnes, C. to G., Newbury. May 27.

ASSISTANT NURSE (£25), Hartismere Union. H. Warnes, C. to G., Union Offices, Eye, Suffolk. May 17.

ASSISTANT NURSES (£20), Driffield Union. Mr. H. Botterill, C. to G., Great Driffield. May 15.

CHARGE NURSE (£35), Kingston-upon-Hull Guardians. R. H. Winter, C. to G., St. Mary's Chambers, Lowgate, Hull. May 14.

CHARGE NURSE (£30), Rochford Union. Mr. F. Gregson, C. to G., Southend-on-Sea. May 13.

CHARGE NURSE (£30), Bucklow Union. The C. to G., Knutsford. May 13.

CHARGE NURSE (£30), Exeter Guardians. Mr. A. Snell, C. to G., Exeter. May 15.

CHARGE OR STAFF NURSE (£35), Macclesfield Union. F. May, C. to G., Union Offices, Macclesfield. May 13.

CHILDREN'S CARETAKER (£20), Warwick Union. Mr. C. H. Passman, C. to G., Leamington. May 17.

CHILDREN'S NURSE (£30), Merthyr Tydfil Union. F. T. James, C. to G., 134 High Street, Merthyr Tydfil. May 23.

FEMALE ATTENDANT ON INFIRM (£23 18s.), St. George-in-the-East Guardians. Mr. R. M. Lochner, C. to G., Raine Street, Old Gravel Lane, E. May 14.

FOSTER-MOTHER (£30), Steyning Union. Mr. A. Flowers, C. to G., Shoreham-by-Sea. May 13.

FOSTER-MOTHER (£30), Camberwell Guardians. Mr. C. S. Stevens, C. to G., Peckham Road, S.E. May 13.

FOSTER-MOTHER (£25), Wells Union. Mr. A. G. Russ, C. to G., Wells, Somerset. May 21.

GENERAL ASSISTANT TO FOSTER-MOTHER (£22), Sevenoaks Union. Mr. F. H. Vibert, C. to G., Sevenoaks. May 17.

GENERAL ASSISTANT TO MATRON (£20), Reigate Guardians, Mr. F. C. Morrison, C. to G., Reigate. May 14.

HEAD NURSE (£30), Falmouth Union. R. N. Rogers, C. to G., Union Offices, Falmouth. May 15.

HOUSEKEEPER (£22), Warwick Union. Mr. C. H. Passman, C. to G., Leamington. May 17.

INFANTS' ATTENDANT (£25), Reigate Guardians. Mr. F. C. Morrison, C. to G., Reigate. May 14.

JUNIOR ASSISTANT NURSE (£15), Malling Union. F. J. Allison, C. to G., West Malling, Kent.

LABOUR MASTER (£30), Fulham Guardians. Mr. E. J. Mott, C. to G., 129 Fulham Palace Road, W. May 21.

LABOUR SUPERINTENDENT (£30), Whitehaven Union. Mr. E. B. Croasdell, C. to G., Whitehaven. May 13.

MALE ATTENDANT (18s. weekly, non-resident), Greenwich Union. Mr. S. Saw, C. to G., Greenwich. May 20.

MALE ATTENDANT (£30), Fulham Guardians. Mr. E. J. Mott, C. to G., 129 Fulham Palace Road, W. May 21.

MALE IMBECILE ATTENDANT (£30), Prestwich Union. Mr. E. W. Ogden, C. to G., Cheetham Hill Road, Manchester. May 13.

MALE NIGHT NURSE (15s. weekly), Croydon Union. Mr. H. List, C. to G., Mayday Road, Thornton Heath, Surrey. May 18.

MALE NURSE (£40), Fulham Guardians. Mr. E. J. Mott, C. to G., 129 Fulham Palace Road, W. May 21.

MATRON (£90), West Ham Union. Mr. T. Smith, C. to G., Leytonstone, N.E. May 22.

MATRON (Foster-Mother) (£40), Sevenoaks Union. Mr. F. H. Vibert, C. to G., Sevenoaks. May 17.

NURSE (£30), Isle of Thanet Union. Mr. C. Taylor, C. to G., Minster, near Ramsgate. May 20.

NURSE (£30), Barnet Union. A. Wilshire, C. to G., Union Offices, Barnet. May 13.

NURSE (£30), Knighton Union. E. H. Deacon, C. to G., Union Offices, Knighton. May 15.

NURSES (2) (£25 or £30 according to qualifications), Winchester Union. Mr. F. Faithfull, C. to G., Winchester. May 13.

PORTER (£28), Sevenoaks Union. Mr. F. H. Vibert, C. to G., Sevenoaks. May 17.

PROBATIONER (£14), Worcester Union. Mr. J. G. Sheild, C. to G., Worcester. May 13.

PROBATIONERS (£10), Hackney Union. Matron of the Infirmary, High Street, Homerton, N.E.

RELIEF FOSTER-MOTHER (£30), Camberwell Guardians. Mr. C. S. Stevens, C. to G., Peckham Road, S.E. May 13.

SICK WARD NURSES (£33), Poplar Guardians. Mr. G. H. Lough, C. to G., Upper North Street, Poplar, E. May 13.

STAFF NURSE (£22), Camberwell Guardians. Mr. C. S. Stevens, C. to G., Peckham Road, S.E. May 13.

STOCKTAKER (£25), Poplar Guardians. Mr. G. H. Lough, C. to G., Upper North Street, Poplar, E. May 13.

TAILOR (£30), Steyning Union. Mr. A. Flowers, C. to G., Shoreham-by-Sea. May 13.

TAILOR and FOSTER MOTHER (married couple) (£30 each), Steyning Union. Mr. A. Flowers, C. to G., Shoreham-by-Sea. May 13.

THIRD-CLASS CLERK (£80), Lambeth Guardians. Mr. J. L. Goldspink, C. to G., Brook Street, Kennington Road, S.E. May 13.

WARDSMAID (£18), St. Ives Union. Mr. G. D. Day, C. to G., St. Ives, Hunts. May 13.

### Institutional and Administrative Vacancies.

ASSISTANT MATRON (£40), Preston Royal Infirmary. W. Davies, Secretary, 5 Winckley Street, Preston. May 13.

LADY DISPENSER (£30), Ross-on-Wye Cottage Hospital. Secretary. May 14.

MATRON (£100), City of London Hospital for Diseases of the Chest, Victoria Park, E. G. Watts, Secretary. May 13.

MATRON (£50), High Wycombe Cottage Hospital. Secretary, Havenfield, High Wycombe.

### Books for Institutional Workers.

| | |
|---|---|
| "Midwife's Pocket Dictionary, with revised Rules of the C.M.B." ... | 1s. 1d. |
| "The Nurses' Pronouncing Dictionary" ... | 2s. 0d. |
| "A Handbook for Nurses." (J. K. Watson, M.D.) | 5s. 4d. |
| "Art of Feeding the Invalid." ... | 1s. 6d. |
| "Surgical Ward Work and Nursing." ... | 5s. 5d. |
| "Massage Movements, including the Nauheim Exercises" ... | 1s. 1d. |
| "Surgical Instruments and Appliances used in Operations" (Harold Burrows, M.B.London, B.S., F.R.C.S.) ... | 1s. 8d. |
| "The Nurse's Materia Medica" (Herbert French, M.D.) ... | 2s. 9d. |

Case-papers, Charts, Professional Cards, and every kind of Institutional Literature.

Of all Booksellers or of The Scientific Press, Limited, 28 and 29 Southampton Street, Strand, London, W.C.

